# Mass evacuation of victims from emergency areas by medical modules aboard the aircraft of EMERCOM of Russia

**DOI:** 10.1186/cc11088

**Published:** 2012-03-20

**Authors:** I Yakirevich, A Popov

**Affiliations:** 1 EMERCOM of Russia, Zhukovsky, Moscow Region, Russia

## Introduction

During elimination of medical consequences of various emergencies the issues concerning victims' mass evacuation to a specialized hospital base are constantly brought up. The physicians of the Central Airmobile Rescue Service of EMERCOM of Russia and the specialists of Kazan Helicopter Plant 'Zarechye' developed two types of modules. The Medical Airplane Module (MMS) is used for medical evacuation of four victims aboard Ilyushin 76 aircraft. The Medical Helicopter Module (MMV) is used for medical evacuation of two victims aboard an MI 8 helicopter. MMS and MMV advantages are: mobility - the possibility of installation in various aircraft cabins types; and versatility - the possibility of any required equipment installation for the treatment of victims with various trauma severity, safe fixation of medical equipment straight on the module, equipment operation off-line as well as using the aircraft power supply network.

## Methods

From December 2008 until now 28 medical evacuations were carried out using MMS aboard Iluyshin 76 aircraft: traffic accident victims, terrorism act victims and manmade catastrophes. In total, 198 patients were evacuated (including 12 children), 55 victims with artificial lung ventilation (ALV). Medical evacuation of severely injured children and adults from regional hospitals to Moscow specialized hospitals in order to provide efficient and modern medical aid was carried out using MMV. In total, 27 patients were evacuated (including five children), five patients with ALV. The majority of victims were in severe and extremely severe conditions with associated multisystem trauma. Closed craniocerebral injury was observed in 75% of victims with mass affection of locomotor apparatus, mine and explosion trauma, gunshot wounds, burn shock and burn disease. Constant monitoring, oxygen therapy, ALV, analgesia and sedation, intensive and anti-shock care as well as wound dressing were carried out in flight. The victims' general condition was evaluated according to the Glasgow Coma Scale, APACHE II and SOFA scales.

## Results

MMS and MMV application in case of mass evacuation in flight ensures spare victims' transportation, total monitoring and treatment continuity. It enables one to carry out anesthetic and resuscitation treatment, intensive care, monitoring and treatment of all the victims.

## Conclusion

The quality of mass medical evacuation of extremely injured victims has considerably improved and the time of transportation from emergency area to specialized hospitals to render them efficient medical aid has reduced.

